# In memoriam: Prof. Dr. rer. nat. Dr. med. h.c. Lore Zech; 24.9.1923 – 13.3.2013: Honorary member of the European Society of Human Genetics, Honorary member of the German Society of Human Genetics, Doctor laureate, the University of Kiel, Germany

**DOI:** 10.1186/1755-8166-6-20

**Published:** 2013-05-21

**Authors:** Brigitte Schlegelberger

**Affiliations:** 1 Institute of Cell and Molecular Pathology, Hannover Medical School, Carl-Neuberg-Str. 1, Hannover D-30625, Germany

## 

**Figure 1:**
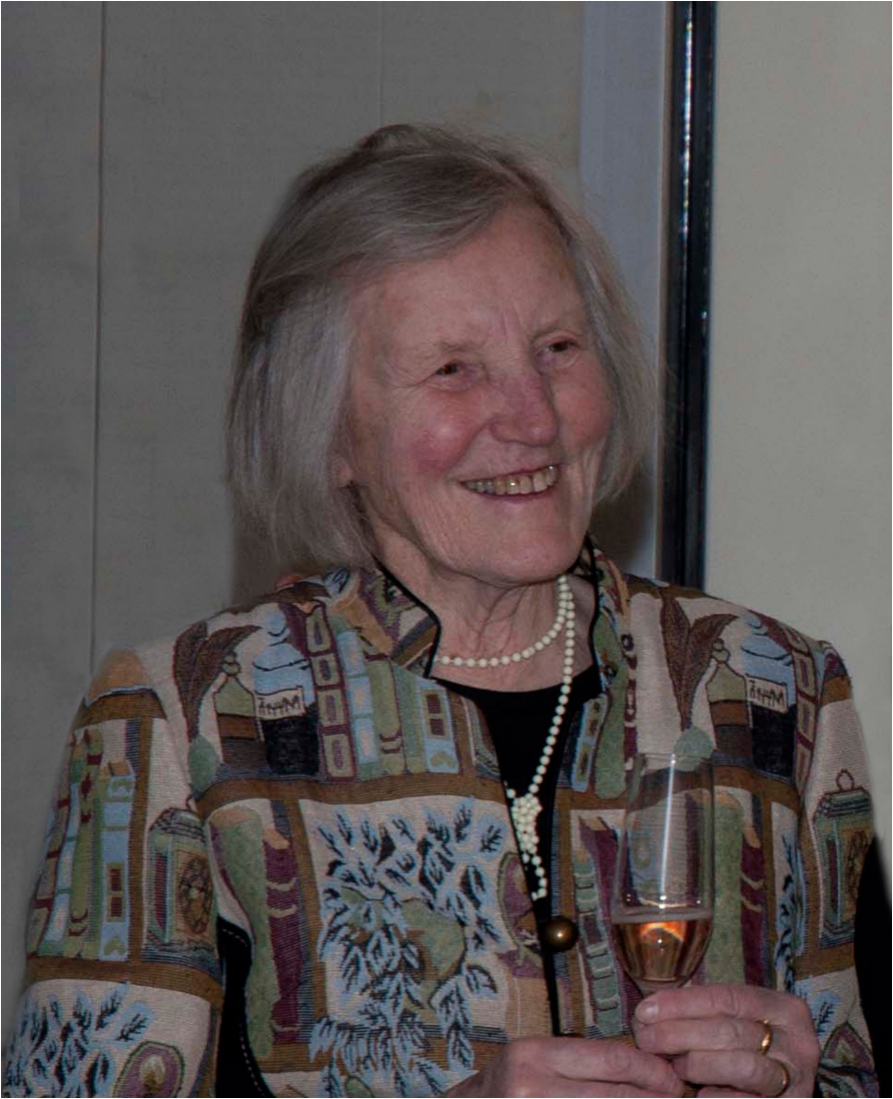
Dr. Lore Zech as we knew her.

Lore Zech (Figure 1) was in a certain way the mother of modern cytogenetics. Without her major contribution, i.e. the development of the first chromosome banding technique to differentiate human chromosomes, modern cytogenetics would not have become the important tool for clinical and tumor cytogenetics it is today. During the 1960s, Lore Zech worked with Torbjörn Caspersson (15.10.1910-07.12.1997) in the Institute of Cell Research and Genetics located at the famous “Karolinska Institutet” in Stockholm, related to Alfred Nobel and his Nobel Prize. Using DNA-binding fluorescence dyes like quinacrine mustard, chromosome banding of plant chromosomes had already been developed there. However, Lore Zech was the one who was convinced that human chromosomes also have different bands, an idea that was not supported by the department head at that time. Working in secret in a small chamber, she developed the so-called Q-banding technique. With her special humor she used to tell that the breakthrough came when she was able to study “valuable male blood”, where she recognized a bright chromosome band, the heterochromatic region of the Y-chromosome [1,2].

In 1971, during the IVth International Congress of Human Genetics in Paris, she was invited to present her findings, and from there on chromosome bands were widely accepted and an international nomenclature established.

During the next decades Lore Zech used the Q-banding technique to identify numerous recurrent chromosome aberrations in human leukemias and lymphomas. The most important example is the Burkitt translocation t(8;14)(q24;q32), still the diagnostic hallmark of Burkitt lymphoma and B-acute lymphoblastic leukemia. She had received tissue of Burkitt lymphomas, which her esteemed collaborator Georg Klein (born 28.07.1925) had obtained from Africa. The description of the Burkitt translocation was the basis to understand that oncogenes are activated by juxtaposition with regulatory sequences, i.e. by translocating the *C-MYC* oncogene into the immunoglobulin heavy chain locus [3,4]. Likewise, the identification of small deletions like the deletion of band 13q14 in B-chronic lymphocytic leukemia (B-CLL) was later recognized to harbor an important tumor suppressor gene [5]. Thus, identification of tumor-specific chromosome aberrations paved the way for the molecular characterization of leukemias and lymphomas and finally for development of targeted therapy [6-15].

Born in Gütersloh, Germany, in 1923, Lore Zech lost both parents at the age of 4 years. She grew up with her grandmother in a rural area, the Sauerland, and began to study Human Medicine in 1944 in Marburg (Germany). After the Second World War, it turned out to be impossible to continue her medical studies and therefore she chose biology, chemistry and physics as her topics in Bonn (Germany). For her doctoral thesis, she went to the Max Planck Institute in Tübingen. In 1953 she followed her husband to Sweden and worked in the Institute of Cell Research and Genetics in Stockholm, until 1989. After her “retirement” she joined the Department of Medical Genetics at the University Hospital of Uppsala, where she spent every day involved in chromosome and FISH analyses as well as discussions with many young colleagues.

Last year, she was extremely proud that a PhD student had dedicated his work to her stating she was his major inspiration. This characterizes her as empathetic and widely interested and someone who stimulated the research of numerous colleagues. When she visited conferences, she was always surrounded by scientists and students, who sought her advice and her company.

One story documents her as a scientist: Lore Zech had detected, but not published, that the Philadelphia chromosome (Ph) was due to the translocation t(9;22)(q34;q11) - she already found that the Ph was a derivative of chromosome 22 [15] - when her American colleague Janet Rowley (born 05.04.1925) visited her and wanted to discuss this finding. She instructed her young colleague how to get better pictures and let her publish this significant finding [16]. Everybody knew that Prof. Dr. Lore Zech was always fair and supportive.

As mentioned above, after her “retirement”, Lore Zech continued to work in and drive every day to the Institute of Medical Genetics, University Hospital of Uppsala, Sweden in her own car. One day, close to her 90^th^ birthday, she was stopped in a traffic control and had a hard time to convince the police officer she was telling the truth when she replied to his question where she was going by: ‘I am going to the institute to work there’.

Lore Zech was loved by her colleagues and, with her wide range of interests she stimulated discussions on many scientific topics. Particularly for students and young investigators, she served as a mentor inspiring and supporting them by sharing her vast skills and knowledge.

Lore Zech died on 13.3.2013 from the progression of chronic lymphocytic leukemia, a disease she had investigated for decades.

Lore Zech is deeply missed by all who had the privilege to meet her.
